# Neuropathology and Therapeutic Intervention in Spinal and Bulbar Muscular Atrophy

**DOI:** 10.3390/ijms10031000

**Published:** 2009-03-10

**Authors:** Haruhiko Banno, Masahisa Katsuno, Keisuke Suzuki, Fumiaki Tanaka, Gen Sobue

**Affiliations:** 1 Department of Neurology, Nagoya University Graduate School of Medicine / 65 Tsurumai-cho, Showa-ku, Nagoya 466-8550, Japan; E-Mails: banno@med.nagoya-u.ac.jp (H.B.); keisuke@med.nagoya-u.ac.jp (K.S.); ftanaka@med.nagoya-u.ac.jp (F.T.); 2 Institute for Advanced Research, Nagoya University / Furo-cho, Chikusa-ku, Nagoya 464-8601, Japan

**Keywords:** Spinal and bulbar muscular atrophy (SBMA), polyglutamine, androgen receptor (AR), leuprorelin acetate

## Abstract

Spinal and bulbar muscular atrophy (SBMA) is a hereditary motor neuron disease caused by the expansion of a polyglutamine tract in the androgen receptor (AR). The histopathological finding in SBMA is loss of lower motor neurons in the anterior horn of the spinal cord as well as in the brainstem motor nuclei. Animal studies have revealed that the pathogenesis of SBMA depends on the level of serum testosterone, and that androgen deprivation mitigates neurodegeneration through inhibition of nuclear accumulation of the pathogenic AR. Heat shock proteins, ubiquitin-proteasome system and transcriptional regulation are also potential targets of therapy development for SBMA.

## 1. Introduction

Polyglutamine diseases are hereditary neurodegenerative disorders caused by an abnormal expansion of a trinucleotide CAG repeat, which encodes a polyglutamine tract. To date, nine polyglutamine diseases are known: Huntington’s disease (HD), spinal and bulbar muscular atrophy (SBMA), dentatorubral-pallidoluysian atrophy (DRPLA) and six forms of spinocerebellar ataxia (SCA) (Table 1). SBMA, also known as Kennedy’s disease, is the first of the neurodegenerative diseases, for which the molecular basis was discovered to be the expansion of a trinucleotide CAG repeat in the gene of the causative gene.

## 2. Clinical and genetic features of SBMA

In general, symptoms of polyglutamine diseases typically appear in mid-life and progressively deteriorate before death from fatal complications. Clinical features vary for each disorder, corresponding to the pathological distribution of neurodegeneration (Table 1).

Major symptoms of SBMA are weakness, atrophy and fasciculations of bulbar, facial and limb muscles [1]. Patients with SBMA occasionally demonstrate signs of androgen insensitivity such as gynecomastia, testicular atrophy, impaired erection and decreased fertility, some of which are detected before the onset of motor impairment. Female carriers are usually asymptomatic, but some express subclinical phenotypes including high amplitude motor unit potentials on electromyography. The progression of SBMA is usually slow, but life-threatening respiratory tract infection often occurs in the advanced stages of the disease, resulting in early death in some patients. The cardinal cause of death is aspiration pneumonia [2].

The molecular basis of SBMA is the expansion of a trinucleotide CAG repeat, which encodes the polyglutamine tract, in the first exon of the androgen receptor (AR) gene [3]. The CAG repeat within AR ranges in size from 11 to 35 in normal subjects, but from 40 to 62 in SBMA patients [3–5]. There is an inverse correlation between the CAG repeat size and the age at onset of motor impairments or the disease severity adjusted by the age at examination in SBMA [6,7] as well as in other polyglutamine diseases [8]. In a nerve conduction study of SBMA, the CAG repeat size and the age at onset were significantly different among the patients with motor- and sensory-dominant phenotypes, indicating that a longer CAG repeat is more closely linked to the motor-dominant phenotype and a shorter CAG repeat is more closely linked to the sensory-dominant phenotype [9].

### 3. Neuropathology and molecular mechanisms of SBMA

The fundamental histopathological finding in SBMA is loss of lower motor neurons in the anterior horn of the spinal cord as well as in the brainstem motor nuclei except for the third, fourth and sixth cranial nerves [10]. The number of nerve fibers is reduced in the ventral spinal nerve root, reflecting motor neuronopathy. Sensory neurons in the dorsal root ganglia are less severely affected, and large myelinated fibers demonstrate a distally accentuated sensory axonopathy in the peripheral nervous system. Muscle histopathology includes both neurogenic and myogenic findings: there are groups of atrophic fibers with a number of small angular fibers, fiber type grouping and clamps of pyknotic nuclei as well as variability in fiber size, hypertrophic fibers, scattered basophilic regenerating fibers and central nuclei.

In general, the abnormal polyglutamine protein forms inclusion bodies in affected neurons, which is a unifying histopathological hallmark of polyglutamine diseases [11]. These neuronal inclusion bodies are often detected in the nucleus, although they may be formed within the cytoplasm or neurites. The deposition of inclusion bodies is not only found in the postmortem neural tissues from patients, but has also been reported in animal models of polyglutamine diseases. The abnormal polyglutamine proteins in the inclusion bodies are often truncated, indicating that proteolytic cleavage appears to enhance the toxicity of the causative gene products [12]. The abnormal polyglutamine proteins are also expressed outside the nervous system, leading to non-neuronal pathology, such as diabetes mellitus, in some polyglutamine diseases [13,14].

In SBMA, nuclear inclusions (NIs) containing the pathogenic AR are found in the residual motor neurons in the brainstem and spinal cord as well as in non-neuronal tissues including the prostate, testes, and skin [15]. These inclusions are detectable using antibodies recognizing a small portion of the N-terminus of the AR protein, but not by those against the C-terminus of the protein. This observation implies that the C-terminus of the AR is truncated or masked upon formation of NI. A full-length AR protein with an expanded polyglutamine tract is cleaved by caspase-3, releasing a polyglutamine-containing toxic fragment, and the susceptibility to cleavage is polyglutamine repeat length-dependent [16]. Thus, proteolytic cleavage is likely to enhance the toxicity of the pathogenic AR protein. Electron microscopic immunohistochemistry shows dense aggregates of AR-positive granular material without limiting membrane, both in the neural and non-neural inclusions, in contrast to the other polyglutamine diseases where NIs take the form of filamentous structures.

A number of studies have indicated that transcriptional dysregulation underlies the molecular mechanism of neuronal dysfunction in polyglutamine diseases. Transcriptional co-activators such as cAMP-response element binding protein-binding protein (CBP) have been shown to be sequestrated into the NIs through protein-protein interaction in mouse models and patients with SBMA [17]. It has also documented that the histone acetyltransferase activity of CBP is inhibited in animal models of polyglutamine diseases, and that histone acetylation level is decreased in a mouse model of SBMA [18]. Taken together, polyglutamine-mediated transcriptional dysregulation appears to play an important role in the pathogenesis of SBMA.

Mitochondrial impairment and oxidative stress have also been stipulated as a causative molecular event in polyglutamine diseases. Depolarization of the mitochondrial membrane and an elevated level of reactive oxygen species have been observed in a cellular model of SBMA [19]. Moreover, the pathogenic AR protein represses the transcription of the subunits of peroxisome proliferator-activated receptor gamma coactivator-1 (PGC-1), a transcriptional co-activator that regulates the expression of various nuclear-encoded mitochondrial proteins [19]. Similar finding have been reported in cellular and animal models of polyglutamine diseases, suggesting that mitochondrial dysfunction is a unifying molecular mechanism whereby abnormal polyglutamine proteins induce neuronal damage.

Obstruction of axonal transport has also gained attention as a cause of neuronal dysfunction in SBMA. The pathogenic AR has been shown to impair axonal transport through a pathway that involves activation of cJun N-terminal kinase (JNK) activity [20]. In a mouse model of SBMA, the nuclear accumulation of the abnormal AR protein induces transcriptional dysregulation of dynactin 1, an axonal motor protein that regulates axonal trafficking [21]. Given that a mutation in the dynactin 1 gene has been shown to cause motor neuron degeneration mimicking SBMA, a disrupted axonal transport is a potential molecular basis for SBMA [22].

## 4. Protein folding abnormalities in SBMA

**Figure 1.**
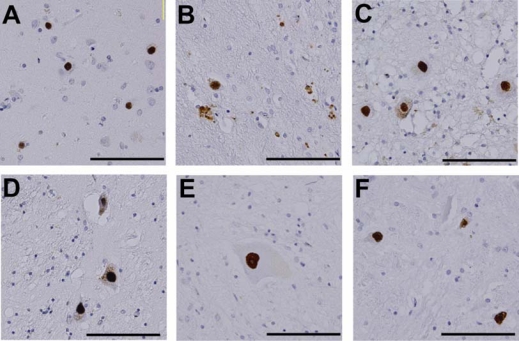
Accumulation of abnormal proteins in polyglutamine diseases. Immunohistochemistry of autopsy specimens from patients using an anti-polyglutamine antibody (1C2). (A) Cerebral cortex, HD; (B) Putamen, HD; (C) Dentate nucleus, DRPLA, (D) Globus pallidus, DRPLA; (E) Anterior horn of spinal cord, SBMA; (F) Pons, SBMA. Scale bar = 100 μm [27].

Although NIs are a disease-specific histopathological finding, their role in pathogenesis has been heavily debated. Several studies have suggested that NIs may indicate a cellular response coping with the toxicity of abnormal polyglutamine protein [23]. Instead, the diffuse nuclear accumulation of the mutant protein has been considered essential for inducing neurodegeneration in polyglutamine diseases including SBMA (Figure 1). Recent data suggest that the toxic species of protein in polyglutamine diseases may be soluble mutant conformers, which can exist as oligomers or monomers containing beta-sheet conformation [24–26].

Although it is difficult to determine the toxic protein species in human histopathology, diffuse accumulation of the causative gene products has been construed as an important finding. An immunohistochemical study on autopsied SBMA patients using an anti-polyglutamine antibody demonstrated that diffuse nuclear accumulation of the pathogenic AR is more frequently observed than NIs in the anterior horn of the spinal cord [28]. Intriguingly, the frequency of diffuse nuclear accumulation of the pathogenic AR in spinal motor neurons strongly correlates with the length of the CAG repeat in the AR gene. No such correlation has been found between NI occurrence and the CAG repeat length. A similar observation has been reported in DRPLA [29]. Taken together, it appears that the pathogenic AR containing an elongated polyglutamine tract principally accumulates within the nuclei of motor neurons in a diffusible form, leading to neuronal dysfunction and eventual cell death in SBMA. In support of this hypothesis, neuronal dysfunction is halted by genetic modulation preventing nuclear import of the pathogenic polyglutamine-containing protein in cellular and animal models of polyglutamine diseases [8].

**Figure 2.**
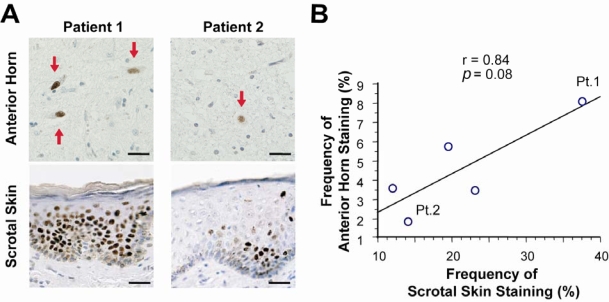
Mutant AR nuclear accumulation in scrotal skin and spinal motor neurons. (A) Mutant AR accumulation was remarkable in both spinal motor neurons and scrotal skin of Patient 1, but less remarkable in both motor neurons and skin in Patient 2. Scale bar = 30 μm. (B) The extent of mutant AR accumulation in scrotal skin epithelial cells showed a tendency to correlate with that in anterior horn cells.

Since the human AR is widely expressed in various organs, nuclear accumulation of the pathogenic AR protein is detected not only in the central nervous system, but also in non-neuronal tissues such as scrotal skin. The degree of pathogenic AR accumulation in scrotal skin epithelial cells tends to be correlated with that in the spinal motor neurons in autopsy specimens, and it is well correlated with CAG repeat length and inversely correlated with the motor functional scale [30]. These findings indicate that scrotal skin biopsy with anti-polyglutamine immunostaining is a good biomarker with which to monitor SBMA pathogenic processes (Figure 2).

## 5. Therapeutic strategies for SBMA

For any given polyglutamine disease, more than one mechanism likely contributes to neuronal dysfunction and eventual cell death. They include: (i) misfolding of the disease protein resulting in altered function; (ii) deleterious protein interactions engaged in by the mutant protein; (iii) formation of toxic oligomeric complexes; (iv) transcriptional dysregulation; (v) mitochondrial dysfunction resulting in impaired bioenergetics and oxidative stress; (vi) impaired axonal transport; (vii) aberrant neuronal signaling including excitotoxicity; (viii) cellular protein homeostasis impairment; and (ix) RNA toxicity [31]. Although each of these molecular mechanisms could be subject to therapeutic interventions, upstream events are more plausible targets than secondary cellular changes.

**Figure 3.**
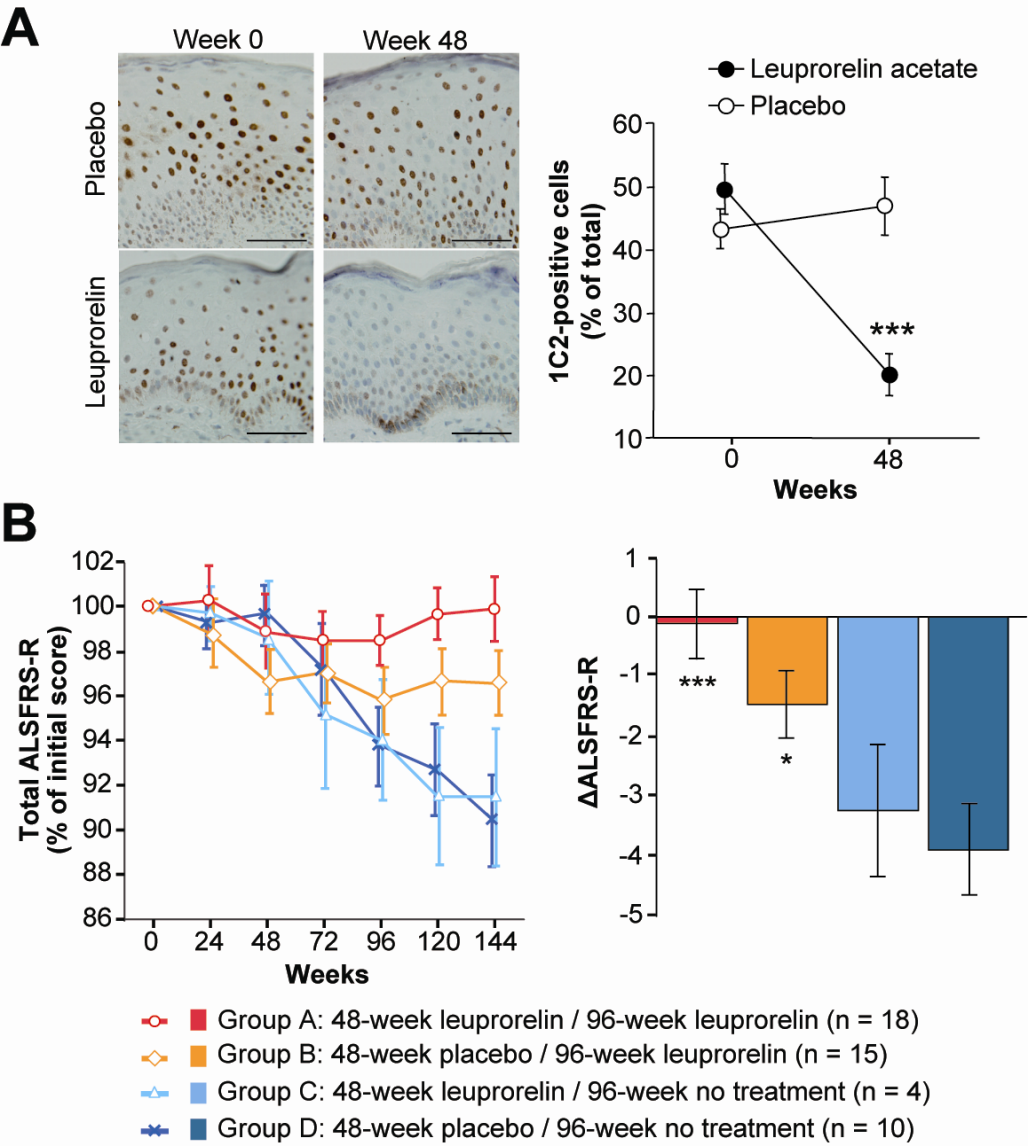
Efficacy results of leuprorelin in SBMA patients. (A) The frequency of diffuse nuclear 1C2 staining (indicative of mutant AR) in the scrotal epithelial cells was significantly decreased after the 48-week administration of leuprorelin acetate. (B) Changes in the ALSFRS-R scores showed treatment duration-dependent improvements in the leuprorelin-treated groups. Scale bars = 50 μm. Data are expressed as means ± SEM. *p < 0.05; **p < 0.005; ***p < 0.001 with respect to Group D [36].

**Figure 4.**
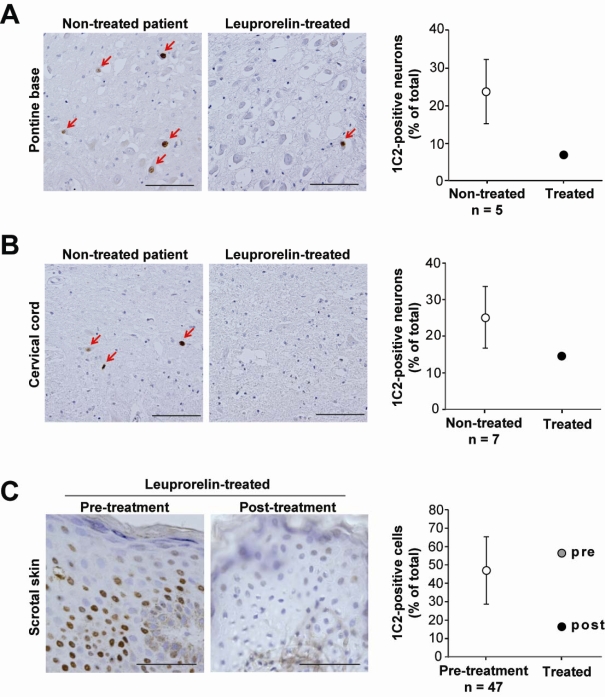
Effects of leuprorelin acetate on nuclear accumulation of mutant AR. (A, B) The accumulation of mutant AR in neurons was remarkable both in the pontine base and in the spinal anterior horn of all the control, non-treated autopsied patients, but the number of 1C2-positive neurons was relatively small in the leuprorelin-treated patient. Scale bars=100 μm. (C) Mutant AR accumulation in biopsied scrotal skin epithelial cells was markedly reduced by leuprorelin. Scale bars=50 μm. Data are expressed as means±SD [36].

There is no well-established disease-modifying therapy for SBMA. Potential therapeutics, however, have emerged from basic research using animal models. Among these therapeutic approaches, androgen deprivation has been translated into clinic [27]. Anti-androgen therapies have been developed taking advantage of the fact that the accumulation of the pathogenic AR proteins is dependent on the circulating level of testosterone [32,33]. Surgical castration has been shown to reverse motor dysfunction in mouse models of SBMA [34]. The luteinizing hormone-releasing hormone analogue, leuprorelin, prevents nuclear translocation of aberrant AR proteins, resulting in a significant improvement of disease phenotype in a mouse model of SBMA [35]. These results of animal studies were verified in a phase 2 clinical trial of leuprorelin, in which the patients treated with this drug exhibited decreased mutant AR accumulation in scrotal skin biopsy, significantly higher functional scores and better swallowing parameters than those receiving placebo (Figure 3). Autopsy of one patient who received leuprorelin suggested that androgen deprivation inhibits the nuclear accumulation and/or stabilization of mutant AR in the motor neurons of the spinal cord and brainstem (Figure 4). These observations suggest that administration of leuprorelin suppresses the deterioration of neuromuscular impairment in SBMA by inhibiting the toxic accumulation of mutant AR [36].

Activation of the cellular defense machinery is another promising therapeutic approach for SBMA. Over-expression of heat shock proteins (HSPs), stress-inducible molecular chaperones, inhibits toxic accumulation of abnormal AR protein and suppresses neurodegeneration in a mouse model of SBMA [37]. Similar beneficial effects have also been achieved by the pharmacological induction of HSPs [38]. On the other hand, inhibition of Hsp90 has been demonstrated to arrest neurodegeneration by activating the ubiquitin-proteasome system in SBMA. Treatment with 17-allylamino geldanamycin (17-AAG), a potent Hsp90 inhibitor, dissociated p23 from the Hsp90-AR complex, and thus facilitated proteasomal degradation of the pathogenic AR in cellular and mouse models of SBMA [39, 40]. Similar effects were observed in the SBMA mice being treated with an oral Hsp 90 inhibitor, 17-(dimethylaminoethylamino)-17-demethoxygeldanamycin (17-DMAG) [41].

Transcriptional dysregulation is another target for therapeutic intervention. Because suppression of histone deacetylase (HDAC) activities results in an augmentation of histone acetylation and a subsequent restoration of gene transcription, HDAC inhibitors have been considered to be of therapeutic benefit in polyglutamine diseases [42]. Butyrate was the first HDAC inhibitor to be discovered, and the related compound, phenylbutyrate, has been successfully employed in experimental cancer therapy. Oral administration of sodium butyrate ameliorates the symptomatic and histopathological phenotypes of a mouse model of SBMA through upregulation of histone acetylation in nervous tissues [18]. This compound has also been shown to alleviate neurodegeneration in a mouse model of DRPLA [43]. In mouse models of HD, the administration of HDAC inhibitors (sodium butyrate, suberoylanilide hydroxamic acid and phenylbutyrate) has been shown to alleviate polyglutamine toxicity and improve neuronal dysfunction [44–46].

## 5. Conclusions

Although the genetics of polyglutamine diseases were discovered as an abnormal expansion of a trinucleotide CAG repeat, detailed mechanisms of the diseases including SBMA have not been fully elucidated. The clinical trial of leuprorelin acetate suggests that androgen deprivation inhibits the nuclear accumulation and/or stabilization of mutant AR in the motor neurons, and thereby stabilizes the disease progression in SBMA patients. Future research approaches have to determine the main mechanisms which contribute to neuronal dysfunction and eventual cell death in SBMA.

## Figures and Tables

**Table 1.** Classification of polyglutamine diseases.

**Disease**	**Major clinical features**	**Affected regions**	**Causative protein**	**Gene (locus)**
Huntington’s disease (HD)	Chorea, cognitive deficits, psychiatric disturbances	Striatum, cerebral cortex	Huntingtin	*IT15* (4p16.3)
Spinal and bulbar muscular atrophy (SBMA)	Weakness, muscular atrophy, bulbar palsy	Spinal cord, brainstem	Androgen receptor	*AR* (Xq13-q12)
Spinocerebellar ataxia type 1 (SCA1)	Ataxia, bulbar palsy, pyramidal signs, muscular atrophy	Cerebellum, brainstem	Ataxin 1	*SCA1* (6p23)
Spinocerebellar ataxia type 2 (SCA2)	Ataxia, slow eye movement, neuropathy	Cerebellum, brainstem	Ataxin 2	*SCA2* (12q24.1)
Spinocerebellar ataxia type 3 (SCA3, Machado-Joseph disease)	Ataxia, bulging eye, parkinsonism, spasticity, fasciculations	Cerebellum, basal ganglia, brainstem, spinal cord	Ataxin 3	*SCA3/MJD* (14q32.1)
Spinocerebellar ataxia type 6 (SCA6)	Ataxia	Cerebellum	α1A-voltage-dependent calcium channel subunit	*CACNA1A* (19p13)
Spinocerebellar ataxia type 7 (SCA7)	Ataxia, retinal degeneration	Cerebellum, retina, brainstem, visual cortex	Ataxin 7	*SCA7* (3p12-p13)
Spinocerebellar ataxia type 17 (SCA17)	Ataxia, cognitive deficits, dystonia, parkinsonism	Cerebellum, striatum	TATA box binding protein	*TBP* (6q27)
Dentatorubral-pallidoluysian atrophy (DRPLA)	Ataxia, myoclonic epilepsy, choreoathetosis, cognitive deficits	Cerebellum, cerebral cortex, globus pallidus, red nuclei, subthalamic nuclei	Atrophin 1	*DRPLA* (12p13.31)
